# Financial Behaviour Under Economic Strain in Different Age Groups: Predictors and Change Across 20 Years

**DOI:** 10.1007/s10603-021-09480-6

**Published:** 2021-03-11

**Authors:** G. Silinskas, M. Ranta, T.-A. Wilska

**Affiliations:** 1 Department of Social Sciences and Philosophy, University of Jyväskylä, Jyväskylä, Finland; 2 Department of Psychology, University of Jyväskylä, Jyväskylä, Finland; 3 Department of Education, University of Helsinki, Helsinki, Finland

**Keywords:** Financial behaviour, Attitudes towards consumption, Cutting expenses, Borrowing, Increasing income, Gambling

## Abstract

The present study examined the multiple micro- and macro-level factors that affect individuals’ financial behaviour under economic strain. The following sociodemographic and economic factors that predict financial behaviour were analysed: age group, year of data gathering, and attitudes towards consumption (economical, deprived, and hedonistic). Subjective financial situations and demographic characteristics were controlled for. Finnish time series data that consisted of five cross-sectional nationally representative surveys were used (*n* = 10 043). The analyses revealed four types of financial behaviour: cutting expenses, borrowing, increasing income, and gambling. Young adults aged 18–25 reported the lowest frequency of borrowing and gambling and the highest frequency of increasing income (together with young adults aged 26–35). Participants aged 66–75 scored the lowest in cutting expenses and increasing income in comparison to all other age groups. Financial behaviour under economic strain in 2019 can be characterized by lower instances of borrowing than in 2004 and 2009 and higher frequencies in increasing income in comparison to all other years of data gathering. Finally, strong attitudes towards saving were related to lower frequency of borrowing and gambling, whereas stronger hedonistic attitudes were related to lower frequency of cutting expenses and more frequent borrowing. The research results provide tools for consumer policy, consumer education, and consumer regulation.

People’s ability to successfully manage their finances relates to their well-being (Dew and Xiao 2013; Serido et al. 2013), and living under economic strain has been suggested to have negative effects on individuals’ mental and physical health. As a consequence, living under economic strain may have adverse effects on the whole society (French and Vigne 2019). Thus, it is important to understand people’s financial behaviour when they face economic difficulties (Baek and DeVaney 2010; Fiksenbaum et al. 2017; Lusardi et al. 2011; Wiersma et al. 2020). Although there is research about consumers’ saving behaviour in different age groups (e.g., Dwyer et al. 2011; Webley and Nyhus 2001), as well as on their attitudes towards credit and debt (e.g., Cloutier and Roy 2020; Gamble et al. 2019; Ottaviani and Vandone 2011), there are too few studies focusing on different kinds of financial behaviour under economic strain (Baek and DeVaney 2010; Lusardi et al. 2011; Wiersma et al. 2020).

Multiple social, demographic, economic, and psychological factors affect the ways individuals use their money in different situations. People exhibit different ways of coping with economic strain, such as cutting expenses (Baek and DeVaney 2010), borrowing (Wiersma et al. 2020), increasing income (Fiksenbaum et al. 2017), and gambling (Lusardi et al. 2011). Financial behaviour depends on income and thus varies by age group. For instance, as young adults are on the verge of gaining financial independence, their low purchasing power makes them cut their expenses, especially when facing financial difficulties (Ranta et al. 2020a). In studies on saving and indebtedness, classical economic theories—permanent income hypothesis (Friedman 1957) and the life cycle theory of savings and consumption (Modigliani and Brumberg 1954)—have been applied. According to these theories, people try to save during their middle age and spend more than their income in their old age. Thus, financial decisions are rational and based on expected future incomes (Friedman 1957; Modigliani and Brumberg 1954).

Theories on financial behaviour as a function of age have been questioned, since many social and psychological factors affect the use of money in different situations. Moreover, people’s behaviour under economic strain is affected by macro-economic conditions (French and Vigne 2019; Hira 2012) including unemployment in particular, but also the availability of credit and loans. Therefore, it is important to examine people’s financial behaviour when under economic strain over a longer time period.

Sociological theories suggest that financial behaviour is relative to peer group behaviour and that the motives for consumption are based on social status (Bourdieu 1984). This classical “keeping up with the Joneses” notion (Dew 2007) may lead to behaviour that might lead to financial difficulties. Also, hedonistic pursuit for pleasure has been noted as a motive for consumption (Campbell 1987). According to the theory of planned behaviour (TPB; Ajzen 1985; Fishbein and Ajzen 2010), attitudes, in particular, shape people’s behaviours, in addition to social and subjective norms, behavioural control, and intention. TPB has been applied to multiple studies on consumer behaviour (e.g., Ajzen 2016; Hegner et al. 2017; Vermeir and Verbeke 2006) as well as to the use of credit and debt (e.g., Cloutier and Roy 2020; Rutherford and De Vaney 2009; Xiao et al. 2011). Thus, in order to understand consumers’ financial behaviour under economic strain, it is important to investigate their attitudes towards consumption.

To our knowledge, there are no studies that systematically illustrate changes in financial behaviour of individuals at different life course stages over a long period of time. To fill this gap, the current study aimed at understanding the multiple micro- and macro-level factors that affect individuals’ financial behaviour under economic strain. Moreover, we focused on different age groups between ages 18 and 75 and across five years of data gathering for 20 years (between 1999 and 2019). The current study was also interested in people’s attitudes towards consumption as well as sociodemographic variables when predicting their financial behaviour.

## Financial Behaviour Under Economic Strain

The most common way of reacting to financial difficulties is cutting current expenses (Fiksenbaum et al. 2017; French and Vigne 2019) and using emergency savings (Baek and DeVaney 2010; Wiersma et al. 2020). For instance, discretionary spending can be cut, and individuals can switch to cheaper substitutes for necessities (Fiksenbaum et al. 2017; Wiersma et al. 2020). The second most popular type of financial behaviour during hardship is borrowing, from either family or friends (French and Vigne 2019; Lusardi et al. 2011; Wiersma et al. 2020), or taking out loans and consumer credit (Gamble et al. 2019; Majamaa et al. 2019; Wiersma et al. 2020). The third type of behaviour relates to activities that increase income (Fiksenbaum et al. 2017; Wiersma et al. 2020), such as working more or selling personal possessions. Another financial behaviour that people report to overcome their financial difficulties with is gambling, that is, trying their luck in various lotteries and games, such as betting, gambling, lotteries, and slot machines (Callan et al. 2008; Lusardi et al. 2011).

Noteworthy, these previous studies analysed financial behaviour related to the individual (Lusardi et al. 2011; Wiersma et al. 2020) or household financial difficulties (Baek and DeVaney 2010; Dew and Xiao 2013) and did not necessarily concern a particular societal context, such as financial boom and bust periods (for exceptions, see Baek and DeVaney (2010) and Dew and Xiao (2013) for the results in response to economic depression of 2007–2009). A systematic review on household financial strain (French and Vigne 2019) confirmed that the strategies people apply on an individual or household level are strikingly similar. Moreover, even though the studies are positioned as taking place at a specific time period (e.g., boom or bust periods), they did not make direct comparisons of the same identical questions assessed at different time periods (e.g., boom vs. bust periods). Consequently, our study was set to fill this gap by directly comparing financial behaviour related to individual financial challenges at different time periods.

In addition, the previous studies varied in the ways they operationalized financial hardships. For example, Lusardi et al. (2011) and Wiersma et al. (2020) asked respondents to evaluate the hypothetical situation of how they would cope with an unexpected 2000 Euro expense in the next month. Baek and DeVaney (2010) asked families retrospectively, whether they experienced any financial difficulties (e.g., income shortfall) during the past year and about their reactions to these financial difficulties. Fiksenbaum et al. (2017) asked undergraduate students about their own or their families’ economic hardship in the last few years. They used the Economic Hardship Questionnaire of Lempers et al. (1989), where the 10 items included not only the degree of economic hardship but also some aspects of exact financial behaviour under financial strain (e.g., “Change food shopping or eating habits to save money?”).

In sum, previous studies have operationalized economic strain in a variety of ways and then, in addition, employed measures of financial behaviour to measure people’s reaction to overcome this economic strain. We concentrated on the actual behaviour that our study participants employed in the past to overcome economic strain, without specifying any specific instances of hardship or a timeframe when those instances occurred. By doing this, we expected respondents to report on their temporary financial behaviours, but this did not preclude from some of them reporting on permanent financial behaviours. Moreover, to our knowledge, none of the previous studies asked the same questions at different time points enabling a direct comparison of answers across time. Consequently, subjective evaluations of economic hardship and reporting of the actual financial behaviours in response to those situations across time were in focus of the current investigation.

## Financial Behaviour Under Economic Strain at Different Ages

Financial behaviour amidst financial difficulties depends on people’s age and life course stage (Wiersma et al. 2020; Zick et al. 2012). For instance, young adults face an important transition from parental financial dependence to financial independence (Xiao et al. 2014) at the age of 25–26 or even later (Lee and Mortimer 2009). Young adults are less likely to get loans from banks (in comparison to older adults), because their creditworthiness is lower. They typically have lower salaries and less savings, due to less established careers. Therefore, banks are generally hesitant to offer loans to (young) people with low incomes and credit (Porretta and Santoboni 2014). It can be thus expected that young adults must cut their expenses and try to increase their income more than other age groups, who have better access to credit. For example, in the study of Ranta et al. (2012) of Finnish youth on the verge of independence at age 25, experienced economic pressure was indicated by having to make cutbacks in expenditures, namely postponing large purchases, decreasing clothing expenses, and using long-term savings for daily expenditures, as opposed to using credit and taking loans. In terms of gambling, studies report that young adults (aged 18–24) are more likely to experience gambling problems than other age groups (Wiebe et al. 2006), and overall gambling behaviour peaks between ages 22 and 30 (Welte et al. 2011).

At younger (aged 36–45) and mid-middle (aged 46–55) ages, people reach the peak in their careers and income. For many 46–55-year olds, as their children start becoming financially independent, their own consumption habits, preferences, and behaviour under economic strain change. Research shows that confidence in financial decision-making increases with age (Xiao et al. 2015). Thus, management of finances during economic hardship of middle-aged adults may be different than that of young adults and older people. For instance, it has been shown that middle-aged adults are more effective borrowers (paying lower interest rates and fewer fees) than younger and older adults due to their better credit scores and higher incomes (Agarwal et al. 2009). In terms of their financial behaviour under strain, individuals aged 35–55 reported less frequent intentions to borrow from family and friends and less frequent intentions to work more than adults aged 18–34 (Lusardi et al. 2011). Furthermore, adults aged 35–55 used their savings more often than adults aged 18–34 (Lusardi et al. 2011).

Finally, people of older middle age (aged 56–65) or retired (aged 66–75) are often thought to cut their expenses. However, the income and consumption levels of people aged 55–65 have risen in the last 30 years (Atkinson and Hayes 2010). Thus, the alternative possibility that older people do not reduce their consumption may also be true, as some people of this age may have more possibilities to enjoy their financial savings. Studies show that people older than 55 were less likely to borrow from family and friends in comparison to younger individuals (Wiersma et al. 2020). Moreover, individuals aged 55–64 were more likely to rely on savings in comparison to those aged 18–34 (Lusardi et al. 2011) and were less likely to sell their possessions (e.g., homes, investments) when under economic strain in comparison to individuals younger than 55 (Wiersma et al. 2020). Interestingly, in comparison to young adults (aged 18–34), individuals aged 55–65 were less likely to use alternative ways of getting loans (e.g., quick loans, pawning own assets), sell things, or work more when facing financial difficulties (Lusardi et al. 2011). Clarifying these expected patterns of financial behaviour when facing financial difficulties by comparing different age groups was one of the main goals of the present study.

## Financial Behaviour Under Economic Strain in Changing Times in Finland Across 20 Years

The macro-economic situation affects individuals’ financial behaviour (Conger et al. 2010). Finland experienced an economic depression in 1990–1994 and an economic boom in the late 1990s and early 2000s. The economic depression caused financial strain to many people, particularly young adults. There was mass unemployment and major cuts in social security benefits (Wilska 1999). Interest rates were high, and many people became severely indebted. Credit taking expanded due to the deregulation of the financial market (Jonung et al. 2008). In the latter part of the 1990s, the economy recovered and the early 2000s witnessed a new boom, after a short recession caused by the internet bubble (overvalued IT-related companies) burst in 2001. The dynamics of bust and recovery affected people’s economic resources, credit taking, consumer behaviour, and also attitudes towards consumption (Autio and Heinonen 2004; Räsänen 2003; Wilska 2002).

The expenditures of Finnish households increased steadily until the finance crisis in 2008–2009. The crisis changed people’s consumer behaviour globally, and many consumers faced economic strain (Fiksenbaum et al. 2017; French and Vigne 2019; Hira 2012). The recovery from the financial crisis was slow in Finland. Although there was small annual growth in GDP during most years from 2010 until the COVID-19 crisis in 2020, there was not a similar economic boom in the early 2000s. Individual consumption increased only slowly in the latter part of the 2010s.^1^

Taking the time period into account is also important in terms of the supply of loans and credit. In Finland, taking consumer credit started to increase during the economic boom of the 2000s. Instant loans provided by private companies were introduced in 2005, and in the beginning, there were no regulations for granting loans (e.g., Raijas et al. 2010). Particularly, young people with low income were target groups of the instant loan−providing companies (Autio et al. 2009). Later, during the 2010s, the regulation for granting instant loans and their interest rates became gradually stricter in Finland. Consequently, in 2019, the instant loan provision had turned into private nonguaranteed consumer credits, credit accounts, and lines of credit of both banks and private companies outside of financial regulation. The total amount of consumer debt increased steadily during the entire 2010s in Finland (Raijas 2019).

As described above, the effects of economic fluctuations and its social consequences on people’s financial behaviour depend heavily on their age. For example, young adults with low income or lack of work experience are economically vulnerable in many ways and may also experience an economic recession more dramatically than older generations (Gesthuizen and Scheepers 2010; Ranta et al. 2020b; Wilska 1999). Thus, we explored the interaction between the time of data gathering and the age group on people’s financial behaviour when under economic strain.

## Financial Behaviour and Attitudes Towards Consumption

Consumption—spending money on any goods and services that consumers perceive necessary, desirable or enjoyable—is an essential part of financial behaviour. The motives for consumption and perceptions of necessities vary greatly between individuals (Aro and Wilska 2014). Measuring attitudes towards consumption is thus important when explaining financial behaviour under economic strain. In many studies, TPB (Ajzen 1985; Fishbein and Ajzen 2010) is utilized to explain financial behaviour by attitudes, norms, intentions, and behavioural control. In studies of consumer behaviour, attitudes towards consumption that reflect both personal preferences and social pressures have been found to explain numerous kinds of consumption (e.g., Ajzen 2016; Hegner et al. 2017; Vermeir and Verbeke 2006). In this study, three types of attitudes towards consumption and financial behaviour can be encapsulated: saving-oriented, deprived, and hedonistic (Kuoppamäki et al. 2017; Wilska 2002). The saving-oriented attitude is characterized by behaviour that avoids borrowing and saves for purchases or unexpected future events. It could be expected that the saving-oriented attitude would be related to more frequent cutting of expenses and lower borrowing. The deprived attitude towards consumption is characterized by the feeling of inability to spend money on the things a person likes. This attitude may also be related to a higher prevalence of cutting expenses and avoiding borrowing. Finally, the hedonistic attitude towards consumption can be characterized by the prevalence of impulsive shopping and overall enjoyment of the consumption (Kuoppamäki et al. 2017). This attitude towards consumption is related to less frequent cutting of expenses and more frequent borrowing.

## Research Questions

The goal of the current study was to investigate people’s financial behaviour under economic strain and to predict this financial behaviour by age group, year of data gathering, and attitudes towards consumption. To achieve these aims, we used Finnish time-series data derived from surveys that collected representative samples every 5 years between 1999 and 2019. The research questions were:

RQ1: To what extent does financial behaviour under economic strain differ by the age groups (ages 18–25, 26–35, 36–45, 46–55, 56–65, and 66–75)?

RQ2: How has financial behaviour under economic strain changed across the last 20 years (1999, 2004, 2009, 2014, and 2019)?

RQ3: To what extent do attitudes towards consumption (saving-oriented, deprived and hedonistic) predict financial behaviour under economic strain?

Previous research has identified several other important factors that may explain financial behaviour under economic strain; thus, we controlled their effects in our analyses. One of these factors, subjective evaluation of the current financial situation, has been put forward as an important determinant of financial behaviour during economic hardship (Fiksenbaum et al. 2017; Ranta and Salmela-Aro 2018). Also, relations have been found between financial behaviour and demographic characteristics, such as gender, place of living, education, and personal income (Wiersma et al. 2020; Xiao et al. 2015; Zick et al. 2012). For instance, females are less likely to borrow than males (Wiersma et al. 2020). Higher educated individuals are less likely to sell their personal belongings (Wiersma et al. 2020) and take out credit from banks (Lusardi et al. 2011).

## Method

### Participants and Procedure

The data was derived from a set of five cross-sectional surveys “Finland—Consumption and Lifestyle”. The purpose of the survey was to follow changes in people’s consumption and other financial behaviours, attitudes, and lifestyles in the beginning of the new Millennium. During this time in Finland, a new ICT-driven economic boom was causing major changes in lifestyles of people who were still recovering from a deep economic depression. The survey was first carried out in 1999 (*n* = 2366) and repeated every five years, in 2004 (*n* = 3458), 2009 (*n* = 1165), 2014 (*n* = 1318), and 2019 (*n* = 1736). The five-year gap was evaluated to be long enough to detect changes in consumers’ attitudes. Each year, the questionnaires were sent to new participants (aged 18–74). Participants were selected from the Finnish Population Register Database using stratified random sampling where the population was stratified by age. The response rate was 61%, 60%, 49%, 46%, and 44%, in the years 1999, 2004, 2009, 2014, and 2019, respectively (Kuoppamäki et al. 2017; Saari et al. 2019). The final data set of *n* = 10 043 was corrected by weighing the data by age and gender.

### Measures

Identical statements were used in all data collections (1999, 2004, 2009, 2014, and 2019). Descriptive statistics are presented in Table 1 and correlations in Table 2.

**Table 1:** Descriptives of all study variables

Variable	*n* (%)	*M*	*SD*	*α*	Range		Skewness
					Potential	Actual	
Year of data gathering	10 258 (100%)						
1999	2492 (24.3%)						
2004	3448 (33.6%)						
2009	1202 (11.7%)						
2014	1351 (13.2%)						
2019	1765 (17.2%)						
Age group	10 016 (100%)						
Ages 18–25	1342 (13.4%)						
Ages 26–35	1767 (17.6%)						
Ages 36–45	1901 (19.0%)						
Ages 46–55	2036 (20.3%)						
Ages 56–65	1752 (17.5%)						
Ages 66–75	1217 (12.2%)						
Financial behaviour							
Cutting expenses	10 114	3.72	0.89	.71	1–5	1–5	− 0.63
Borrowing	9937	1.64	0.76	.66	1–5	1–5	1.50
Increasing income	9863	2.19	1.09	.34	1–5	1–5	0.72
Gambling	9912	2.02	1.12		1–5	1–5	0.89
Attitudes towards consumption							
Saving-oriented	10 200	3.71	0.75	.56	1–5	1–5	− 0.29
Deprived	10 153	2.94	1.14	.61	1–5	1–5	0.08
Hedonistic	10 167	2.74	0.77	.42	1–5	1–5	0.06
Subjective financial situation	10 185	3.32	0.82		1–5	1–5	− 0.35
Gender (1 = male, 2 = female)	10 117 (100%)	1.50	0.50		1–2	1–2	− 0.01
Male	5045 (49.9%)						
Female	5072 (50.1%)						
Place of living (1 = urban, 2 = rural)	9966 (100%)	1.23	0.42		1–2	1–2	1.31
Urban	7712 (77.4%)						
Rural	2255 (22.6%)						
Education	9587 (100%)	3.19	1.67		1–7	1–7	0.43
Personal income (NETTO/month)	9351	1523.76	1723.53			0–80 000	17.19

**Table 2:** Correlations between all study variables

		1	2	3	4	5	6	7	8	9	10	11	12
1	Age group												
2	Cutting expenses	− .061**											
3	Borrowing	− .027**	− .006										
4	Increasing income	− .211**	.192**	.184**									
5	Gambling	.081**	.063**	.227**	.061**								
6	Subjective financial situation	.035**	− .182**	− .198**	− .093**	− .171**							
7	Attitudes: saving-oriented	.202**	.284**	− .252**	− .008	− .014	− .008						
8	Attitudes: deprived	− .030**	.220**	.213**	.132**	.247**	− .474**	.064**					
9	Attitudes: hedonistic	− .206**	− .021*	.085**	.077**	.092**	.022*	− .099**	.130**				
10	Gender (1 = male, 2 = female)	.036**	.175**	− .070**	− .109**	− .066**	− .001	.024*	.017	.037**			
11	Place of living (1 = urban, 2 = rural)	.124**	− .004	− .022*	.017	.019	− .035**	.085**	.041**	− .064**	− .022*		
12	Education	− .107**	.012	− .022*	.004	− .181**	.234**	− .122**	− .214**	.006	.089**	− .152**	
13	Personal income	.069**	− .085**	.001	.004	− .032**	.221**	− .052**	− .128**	− .015	− .106**	− .032**	.201**

*p < .05; **p < .01

#### Age Groups

We asked participants to report their age in years. We categorized responses into six age groups: 18–25, 26–35, 36–45, 46–55, 56–65, and 66–75.

#### Financial Behaviour Under Economic Strain

The measure was similar to the one used in previous studies (Fiksenbaum et al. 2007; Kuoppamäki et al. 2017; Lusardi et al. 2011). Participants were asked a question (“What have you done when you have been in a financially tight situation?”) and presented 10 statements representing behaviour during economic hardship. Each statement was evaluated on a five-point Likert scale, with values ranging from 1 (“Never”) to 5 (“Often”). We ran principal axis factor analysis with Oblimin rotation and, based on eigenvalue (>1), obtained three factors: cutting expenses, borrowing, and increasing income (Table 3). Mean scores were calculated for each dimension of the financial behaviour scale. The item “I tried luck at gambling games” did not load on any three extracted factors (loadings for all factors were lower than .30). Therefore, we analysed it separately as the fourth dimension “gambling”.

**Table 3:** Factor loadings of the principal axis factor analysis for financial behaviour under economic strain and attitudes towards consumption

Financial behaviour when under economic strain		Cutting expenses	Borrowing	Increasing income
What have you done if you have been in a financially tight situation?				
1.	Additional work done			.317
2.	Bought cheaper food	.732		
3.	Avoided borrowing	.560		
4.	Borrowed from friends		.343	
5.	Has taken out a consumer credit		.853	
6.	Has taken out a further loan from a bank		.547	
7.	Reduced all spending and made a living simply	.710		
8.	Tried luck at gambling games			
9.	Tried to take advantage of special offers	.691		
10.	Sold my possessions			.516
	Eigenvalue, % (cumulative %)	18.233 (18.233)	10.163 (28.396)	2.736 (31.131)
	Cronbach’s alpha (*α*)	.710	.660	.337
Attitudes towards consumption		Saving-oriented	Deprived	Hedonistic
1.	Everyone should save for a rainy day	.529		
2.	I find myself living frugally	.504		
3.	I fund my purchases by saving in advance	.605		
4.	Borrowing should be avoided	.344		
5.	Most of my problems would be solved if I had more money at my disposal		.696	
6.	I feel that I must not spend on things I want, but I must compromise a lot on my own needs for my family		.631	
7.	I often do impulsive shopping			.539
8.	I want to enjoy my consumption			.444
9.	What matters to me is what other people think of me			.342
	Eigenvalue, % (cumulative %)	12.954 (12.954)	11.252 (24.206)	5.818 (30.024)
	Cronbach’s alpha (*α*)	.561	.609	.420

#### Attitudes Towards Consumption

The measure was similar to the one used in previous studies (e.g., Aro and Wilska 2014; Kuoppamäki et al. 2017; Räsänen 2003; Wilska 2002). Participants were presented nine statements concerning attitudes towards consumption. They evaluated each statement on a five-point Likert scale, with values ranging from 1 (“Completely disagree”) to 5 (“Completely agree”). Principal axis factor analysis with Oblimin rotation was performed for the nine items (Table 3), and three factors were extracted based on eigenvalues (>1): saving-oriented, deprived, and hedonistic consumption. Mean scores were calculated for each attitude.

#### Subjective Financial Situation

was measured by asking one question (“How would you describe your financial situation at the moment?”). A five-point Likert scale was used, with values ranging from 1 (“Very bad”) to 5 (“Very good”).

#### Demographic Characteristics

Participants answered questions concerning their gender, place of living, highest level of education, and personal income (net income/month).

### Analysis Strategy

To investigate our research questions, we ran analysis of covariance (ancova) by using the univariate general linear model in SPSS. We ran four separate models for each dimension of financial behaviour (cutting expenses, borrowing, increasing income, and gambling) as the dependent variables. For each model, the independent variables included age group (Age; 18–25, 26–35, 36–45, 46–55, 56–65, and 66-75), year of measurement (Year; 1999, 2005, 2009, 2014, and 2019), and the interaction term of the two—Age × Year. Other predictors included attitudes towards consumption (saving-oriented, deprived, and hedonistic) and control variables: subjective financial situation, gender, place of living, highest level of education, and personal income. In all four univariate general linear models, the overall statistical significance of the independent variables was indicated by the *F* values and their significance level (*p*). We have also reported the total explained variance (adj. *R*^2^) for the overall model and the unstandardized parameter estimate (*B*) and partial eta-square (partial *η*^2^) for individual predictors. Differences in groups were investigated using Bonferroni comparisons.

## Results

### What Predicts Cutting Expenses?

**Fig. 1:**
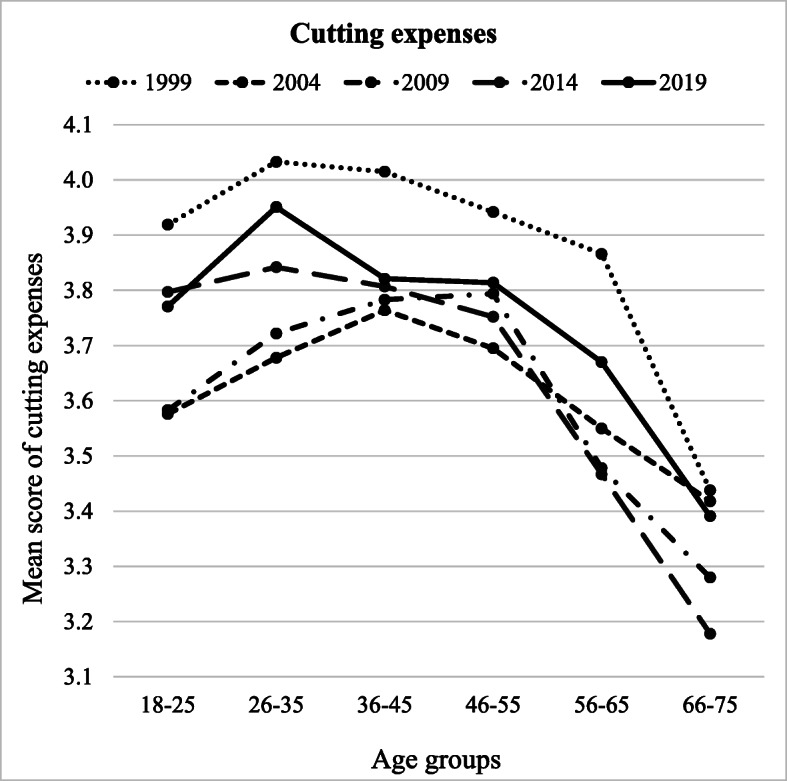
Cutting expenses across different age groups (*x*-axis) and years of measurement (separate lines)

The cutting expenses scale was significantly predicted by age group (*F* (5, 8584) = 56.027, *p* < .001, partial *η*^2^ = .032), year of measurement (*F* (4, 8584) = 31.915, *p* < .001, partial *η*^2^ = .015), and interaction between the two (*F* (10, 8584) = 2.251, *p* = .001, partial *η*^2^ = .005) (Table 4). Participants in the age group 26–35 scored the highest out of all age groups (*M* = 3.85) in cutting expenses and differed from the youngest (18–25; *p* < .01) and the two oldest groups of participants (56–65 and 66–75, *p* < .001). However, they did not differ from the participants in age groups 36–45 and 46–55 (Fig. 1). The age group 66–75 scored the lowest in cutting expenses (*M* = 3.34) and differed from all other age groups (*p* < .001). Cutting expenses was at its highest in 1999 (*M* = 3.87) when it was higher than that in all other years (*p* < .001) (Fig. 1). Cutting expenses was at its lowest in 2009 (*M* = 3.61) and was lower than that in 1999 and 2019 (*p* < .001). However, it did not differ in frequency from 2004 and 2014. Saving-oriented and deprived consumption attitudes were related to higher frequency of cutting expenses, whereas hedonistic consumption attitudes were related to a lower frequency of cutting expenses. Cutting expenses was more common among females (vs. males) and among higher educated people. Furthermore, satisfaction with the current financial situation was negatively associated with cutting expenses.

**Table 4:** Univariate general linear model predicting four financial behaviours under economic strain

Independent variables	Dependent variables																			
	Cutting expenses (adjusted *R*^2^ = .218)					Borrowing (adjusted *R*^2^ = .166)					Increasing income (adjusted *R*^2^ = .101)					Gambling (adjusted *R*^2^ = .123)				
	*B*	*df*	*F*	*p*	Partial *η*^2^	*B*	*df*	*F*	*p*	Partial *η*^2^	*B*	*df*	*F*	*p*	Partial *η*^2^	*B*	*df*	*F*	*p*	Partial *η*^2^
Intercept	2.135	1	583.797	**<.001**	.064	2.541	1	1343.747	**<.001**	.136	2.573	1	359.877	**<.001**	.041	1.748	1	357.338	**<.001**	.040
Gender (1 = male, 2 = female)	.302	1	311.114	**<.001**	.035	− .112	1	55.047	**<.001**	.006	− .234	1	110.016	**<.001**	.013	− .148	1	42.108	**<.001**	.005
Place of living (1 = urban, 2 = rural)	− .023	1	1.193	.275	<.001	− .024	1	1.628	.202	<.001	.100	1	13.351	**<.001**	.002	− .047	1	2.820	.093	<.001
Education	.032	1	33.161	**<.001**	.004	.006	1	1.264	.261	<.001	.009	1	1.451	.228	<.001	− .082	1	121.357	**<.001**	.014
Personal income	<.001	1	2.416	.120	<.001	<.001	1	1.116	.291	<.001	<.001	1	2.042	.153	<.001	<.001	1	0.679	.410	<.001
Subjective financial situation	− .130	1	113.645	**<.001**	.013	− .144	1	181.322	**<.001**	.021	− .075	1	22.368	**<.001**	.003	− .117	1	51.875	**<.001**	.006
Attitudes: saving-oriented	.363	1	931.989	**<.001**	.098	− .260	1	617.271	**<.001**	.068	.030	1	3.872	**.049**	<.001	− .044	1	7.881	**.005**	.001
Attitudes: deprived	.120	1	182.993	**<.001**	.021	.104	1	175.669	**<.001**	.020	.100	1	74.614	**<.001**	.009	.167	1	198.732	**<.001**	.023
Attitudes: hedonistic	− .051	1	18.484	**<.001**	.002	.066	1	39.903	**<.001**	.005	.037	1	5.741	**.017**	.001	.163	1	108.044	**<.001**	.013
Year of data gathering (Year)		4	31.915	**<.001**	.015		4	28.387	**<.001**	.013		4	35.199	**<.001**	.016		4	40.979	**<.001**	.019
Year 1999	.147			.051	<.001	.087			.189	<.001	.245			**.012**	.001	− .048			.630	<.001
Year 2004	− .196			**.004**	.001	.113			.058	<.001	− .479			**<.001**	.003	.378			**<.001**	.002
Year 2009	− .189			**.037**	.001	.101			.204	<.001	− .183			.118	<.001	.143			.234	<.001
Year 2014	.026			.750	<.001	.034			.631	<.001	− .242			**.022**	.001	.132			.223	<.001
Year 2019	0^a^					0^a^					0^a^					0^a^				
Age group (Age)		5	56.027	**<.001**	.032		5	13.212	**<.001**	.008		5	73.512	**<.001**	.042		5	28.297	**<.001**	.016
Age 66–75	− .381			**<.001**	.003	.139			**.031**	.001	− .622			**<.001**	.005	.374			**<.001**	.002
Age 56–65	− .101			.144	<.001	.220			**<.001**	.002	− .464			**<.001**	.003	.548			**<.001**	.004
Age 46–55	.043			.558	<.001	.235			**<.001**	.002	− .322			**.001**	.001	.465			**<.001**	.003
Age 36–45	.050			.504	<.001	.180			**.006**	.001	− .119			.216	<.001	.390			**<.001**	.002
Age 26–35	.180			**.016**	.001	.152			**.020**	.001	− .039			.687	<.001	.193			.051	<.001
Age 18–25	0^a^					0^a^					0^a^					0^a^				
Year × Age		20	2.251	**.001**	.005		20	1.742	**.021**	.004		20	4.855	**<.001**	.011		20	1.862	**.011**	.004
Error	8584					8516					8462					8495				

Values in bold indicate significant results at *p* <.05

^a^This parameter is set to zero because it is redundant

### What Predicts Borrowing?

**Fig. 2:**
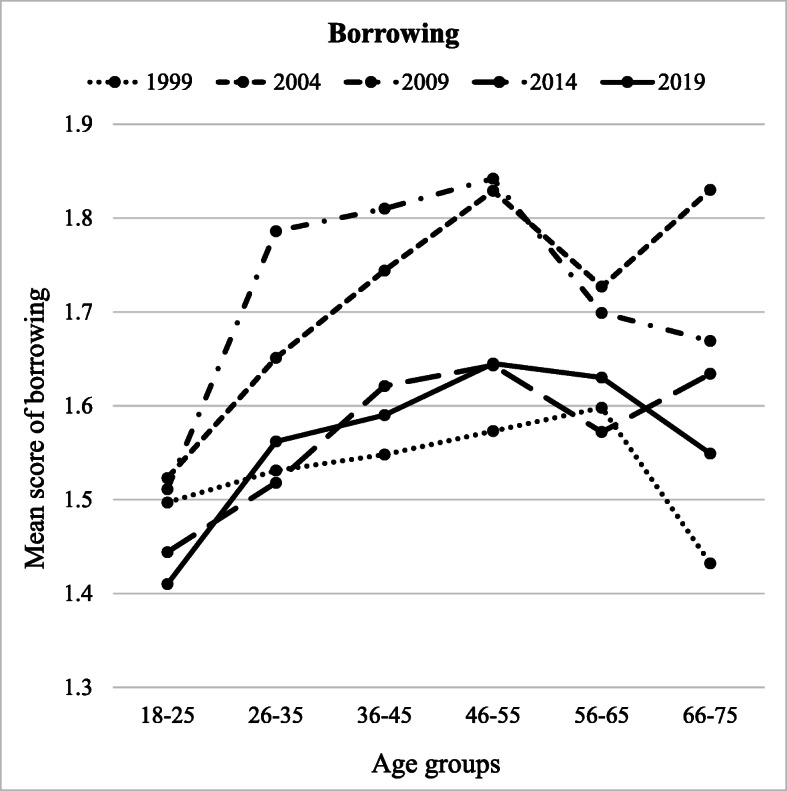
Borrowing across different age groups (*x*-axis) and years of measurement (separate lines)

Borrowing was significantly predicted by age group (*F* (5, 8516) = 56.027, *p* = .008, partial *η*^2^ = .032), year of measurement (*F* (4, 8516) = 31.915, *p* < .001, partial *η*^2^ = .013), and the interaction between the two (*F* (10, 8516) = 2.251, *p* = .021, partial *η*^2^ = .004) (Table 4). The age group of 46–55 scored the highest in borrowing (*M* = 1.71) and differed from both groups of young adults (18–25 and 26–35; *p* < .01), but not from the remaining age groups (36–45, 56–65, and 66–75) (Fig. 2). The youngest age group 18–25 reported the lowest frequency of borrowing (*M* = 1.48) and differed from all other age groups (*p* < .001). Participants in 2004 (*M* = 1.72) and 2009 (*M* = 1.72) borrowed the most and differed from all other years (*p* < .001) (Fig. 2). Participants in 1999 (*M* = 1.53) borrowed the least but did not differ from participants in 2019 and 2014 (*p* < .001). The more participants reported deprived or hedonistic attitudes towards consumption, the more they had borrowed when facing financial difficulties. The stronger the attitudes towards saving, the less borrowing was reported. Borrowing was more common among males (vs. females). Finally, the more people were satisfied with their current financial situation, the less borrowing they reported.

### What Predicts Increasing Income?

**Fig. 3:**
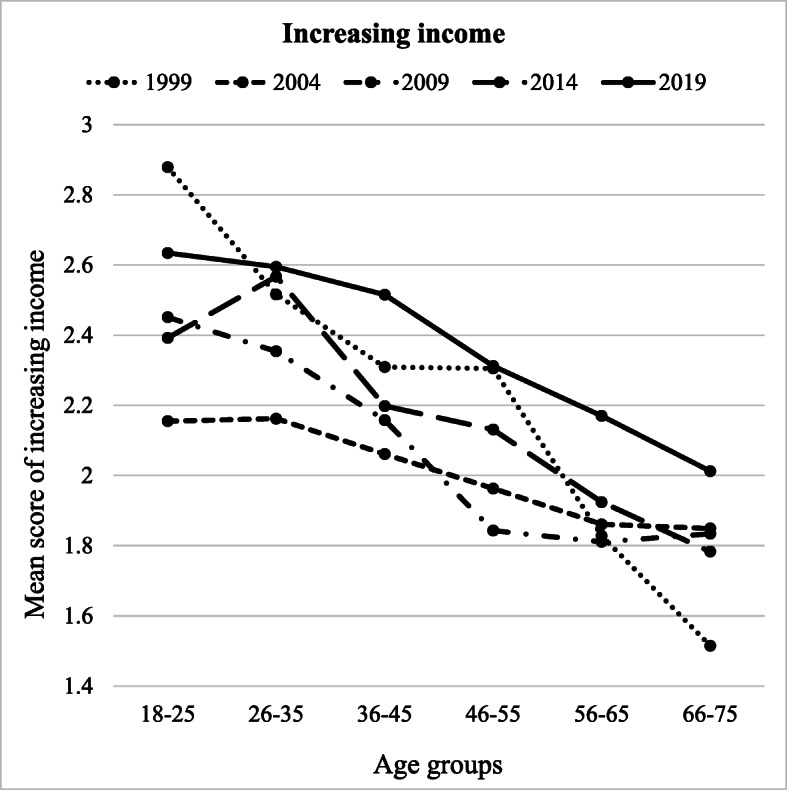
Increasing income across different age groups (*x*-axis) and years of measurement (separate lines)

Increasing income was significantly predicted by age group (*F* (5, 8462) = 73.512, *p* = .042, partial *η*^2^ = .032), year of measurement (*F* (4, 8462) = 35.199, *p* < .001, partial *η*^2^ = .016), and the interaction between the two (*F* (10, 8462) = 4.855, *p* < .001, partial *η*^2^ = .011) (Table 4). Youngest participants (aged 18–25) scored the highest in increasing income (*M* = 2.50) and differed from all other age groups (*p* < .001), except age group 26–35 (Fig. 3). Our oldest participants (aged 66–75) scored the lowest in increasing income (*M* = 1.80) and differed from other groups (*p* < .001), except age group 56–65. Participants in 2019 (*M* = 2.37) reported the highest frequency of increasing income in comparison to all other years of data collection (*p* < .01) (Fig. 3). Increasing income was the lowest in 2004 (*M* = 2.01), and it differed from other years (*p* < .001), except 2009. All three types of attitudes towards consumption were positively related to increasing income: The more participants reported saving oriented, deprived, or hedonistic attitudes towards consumption, the more they reported they were striving at increasing income when facing financial difficulties. Increasing income was more common among males (vs. females) and participants living in rural areas of Finland (vs. urban areas). Also, the more people were satisfied with their current financial situation, the less they reported increasing income.

### What Predicts Gambling?

**Fig. 4:**
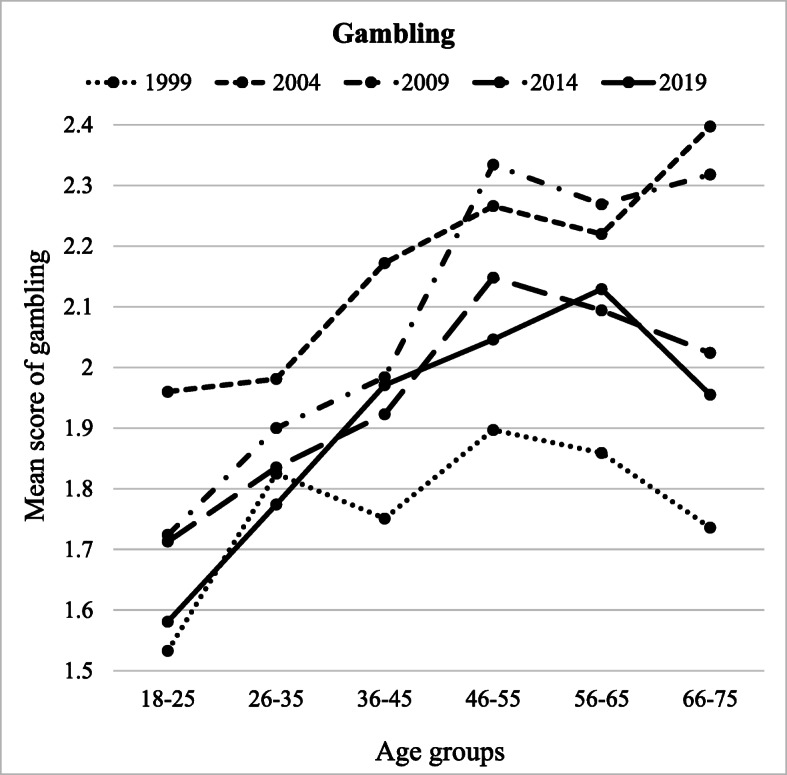
Gambling across different age groups (*x*-axis) and years of measurement (separate lines)

Gambling was significantly predicted by age group (*F* (5, 8495) = 28.297, *p* = .042, partial *η*^2^ = .016), year of measurement (*F* (4, 8495) = 40.979, *p* < .001, partial *η*^2^ = .019), and the interaction between the two (*F* (10, 8495) = 1.862, *p* = .011, partial *η*^2^ = .004) (Table 4). Participants aged 46–45 scored the highest (*M* = 2.14) in gambling and differed from all younger groups (18–25, 26–35, and 36–45, *p* < .001), but not older groups (Fig. 4). The youngest age group reported the lowest frequency of gambling (*M* = 1.71) and differed from all other age groups (*p* < .001). Participants in 2004 (*M* = 2.17) reported higher frequencies of gambling among all groups (*p* < .001) than in other years, except participants in 2009 (Fig. 4). Gambling was the lowest in 1999 (*M* = 1.77) and differed from all other years (*p* < .001). Saving-oriented attitudes towards consumption were related to less frequent gambling, whereas deprived and hedonistic attitudes were related to more frequent gambling. Gambling was more common among males (vs. females) and among lower educated people. Finally, the more satisfied people were with their current financial situation, the less gambling they reported.

## Discussion

The present study investigated people’s financial behaviour under economic strain and its differences depending on participants’ age and year of data gathering between 1999 and 2019. We also examined how attitudes towards consumption affect consumers’ behaviour in economically tight situations. We found four types of financial behaviour: cutting expenses, borrowing, increasing income (working, selling possessions), and gambling. Our analyses revealed that the effects of age, year of data gathering, and attitudes towards consumption on the behaviours varied in interesting ways.

### Financial Behaviour Under Economic Strain in Different Age Groups

The four types of financial behaviour under economic strain varied by participants’ age. Two age groups—the youngest (18–25) and the oldest (66–75)—had the most distinct characteristics in comparison to other age groups. The youngest participants reported the lowest frequency of borrowing and gambling and the highest frequency of increasing income (together with participants aged 26–35). Participants aged 66–75 scored the lowest in cutting expenses and increasing income in comparison to all other age groups.

Participants at the peak of their careers (ages 26–55) reported highest frequencies of cutting expenses under economic strain (not the emerging adults, people approaching retirement, or retired). One explanation may be that consumers at the active stage of their careers with stable income usually spend more than consumers of the other age groups, and thus, they are able to cut expenses when facing economic difficulties. In contrast, people at the very start of their careers might have low income which restricts their consumption, and thus, there is less to cut from (Ranta et al. 2012; Xiao et al. 2014). Interestingly, the most senior participants (aged 66–75) scored the lowest in cutting expenses. On the one hand, people at the retirement age have lower income, and thus, they may minimize their expenses overall. Hence, reducing expenses further becomes impossible. An alternative explanation, which is in line with the permanent income hypothesis (Friedman 1957) and life cycle theory (Modigliani and Brumberg 1954), is that people of this age may have savings, and thus, they do not consider cutting expenses even when under economic strain.

The most frequent borrowers were the participants over the age of 35. Borrowing—both from close people and financial institutions—is common at all stages of adulthood. However, this result contradicts that of Lusardi et al. (2011), who found that individuals aged 18–34 were more likely to borrow from family and friends than older adults, and young adults were more likely to take advantage of consumer credits (e.g., instant loans, pawning the assets) in comparison to individuals aged 55–65. In contrast to the previous studies in the United States (US) (Lusardi et al. 2011) and the Netherlands (Wiersma et al. 2020), we found that, in Finland, borrowing is at its lowest in emerging adulthood (age group 18–25). Therefore, it is possible that Finnish young adults are not yet trusted by banks to be provided with loans. Many young adults are also still financially dependent on their parents, and parental financial support may not be considered borrowing.

As suggested by previous research (Lusardi et al. 2011; Wiersma et al. 2020), we found that the attempts to increase income under economic strain were somewhat linearly dependent on participants’ age. That is, the youngest participants (aged 18–25) scored the highest for increasing income, the oldest participants (aged 66–75) scored the lowest, and the other age groups scored in between. As suggested by the previous literature (Fiksenbaum et al. 2017), our dimension of increasing income included both working more and selling personal goods. Young people scored the highest, suggesting that young people are more likely to have fixed-term work with flexible/irregular hours and a possibility to work more if needed (Lusardi et al. 2011). In contrast, people with established careers and fixed contracts have less flexibility with the amount of work they are able to perform. In addition, the family life of middle-aged adults may restrict them from working more in comparison to emerging adults. In terms of the frequency of selling personal possessions, online-selling opportunities have made the selling of personal items easier and more profitable than before (Lusardi et al. 2011). Arguably, young people are more familiar with the online services and social media and therefore appear to use this option more than other age groups (Silinskas et al. 2021). Older adults, in turn, might value possessing property and feel emotional attachment to the goods obtained throughout the years (Cram and Paton 1993; Gentry et al. 1995).

Finally, participants of our three oldest age groups (46–75) reported more frequent gambling than others, and participants of the youngest age group (18–25) scored the lowest. This was in contrast to previous research which suggests that gambling peaks in youth between the ages 18 and 30 (Welte et al. 2011; Wiebe et al. 2006). However, it is likely that young people gamble for fun rather than trying to solve financial problems.

Our study also investigated the interactions between age and year of data gathering on financial behaviour. A complex pattern of interactions was found (Figs. 1, 2, 3, and 4). However, for clarity reasons, the interactions are easier to understand in the context of the main effects.

### Financial Behaviour Under Economic Strain Across 20 Years (1999–2019)

Our study results highlight the fact that individual behaviour under economic strain is at least partially a reflection of the concurrent macro-environment. For instance, consumption in 1999 was characterized by the higher frequency of cutting expenses, lower instances of borrowing, and less gambling in comparison to any other year of data gathering. Drawdowns of consumer credit were not common in 1999, which can explain less frequent borrowing in 1999 than in other years. Moreover, in 1999, there was an economic boom due to the increasing value of ICT companies, and people’s incomes were growing rapidly. Due to the debt problems caused by the depression in 1990–1994, consumers avoided taking loans for many years and were cautious as consumers. The debt ratio of private households was rather low in 1999 compared to the years before and after.^2^ Another reason is that the deregulation of the financial market in Finland was rather recent, and therefore, financial companies did not have yet a large selection of financial products to offer (Jonung et al. 2008).

Individuals’ financial behaviour under economic strain was somewhat similar in 2004 and 2009. The year 2004 was a period of growing consumer confidence and growth in GDP. Conversely, the year 2009 was still affected by the finance crisis. Yet, both years can be characterized by less frequent instances of cutting expenses and attempts of increasing income (compared to 1999, 2014, and 2019). In contrast, people in 2004 and 2009 relied more on borrowing and gambling than in other years. Although we expected to find this behaviour during the economic boom in 2004, it was surprising that people reported similar behaviour in 2009 during the economic recession. One explanation can be that consumer credit became more freely available, and instant loans were introduced during the economic boom in the 2000s (Autio et al. 2009). Thus, people relied on borrowing to overcome economic difficulties instead of cutting their expenses or increasing income. The debt ratio of Finnish households started to increase steeply around the year 2005,^3^ which indicates both low interest rates but could also reflect changes in attitudes towards credit and debt. On the other hand, due to high levels of unemployment (Dew and Xiao 2013), people in 2009 could have borrowed from other people as they were not able to work more. One more explanation is that people answering the questionnaire in 2009 referred to their previous financial behaviour before the economic recession. Furthermore, the decrease in private consumption in Finland between 2008 and 2009 was not dramatic (Raijas 2014).

Consumers under economic strain borrowed less in the last decade (years 2014 and 2019) than consumers in all previous years of data gathering, which was surprising. The years 2014–2019 were a period of economic growth, and the consumption of private households also increased, albeit slowly. However, the number of the drawdowns of consumer credit has increased notably in recent years, and the debt ratio of private households is higher than ever before.^4^ Therefore, it could be expected that people in economic strain would resort more to borrowing in 2014 and 2019 than before it. It is thus likely that consumers do not always take loans due to financial strain but rather for aspirational purchases (e.g., hedonistic motives). For instance, according to the statistics of the Bank of Finland, the amount of consumer credit typically increases during summer vacation months, which suggests that people require extra money for travelling and other free-time activities.^5^

Consumer behaviour in 2014 was similar to that in 2004 and 2009, with a lower frequency of cutting expenses than that in 1999 and 2019. Finally, financial behaviour under economic strain in 2019 can be characterized by the highest frequency in increasing income in comparison to the other years of data gathering. This can be explained by the good employment situation, as the unemployment rates were lower in 2019 than in previous years.^6^ Moreover, as mentioned above, selling personal goods using online platforms became easier than before.

### Attitudes Towards Consumption

Many studies on financial behaviour have applied the theory of planned behaviour (Ajzen 1985; Fishbein and Ajzen 2010), which suggests that attitudes and intentions are likely to guide people’s financial behaviour (e.g., Ajzen 2016; Hegner et al. 2017; Vermeir and Verbeke 2006). In this study, we found three types of attitudes: saving-oriented, deprived, and hedonistic (Kuoppamäki et al. 2017; Wilska 2002). Saving-oriented attitude towards consumption is primarily related to more frequent cutting of expenses and less frequent borrowing. Hence, it is behaviour that promotes saving (Baek and DeVaney 2010; Lusardi et al. 2011; Wiersma et al. 2020). Similarly, we found that people with higher saving-orientation were more prone to increasing income and were less likely to engage in gambling.

The deprived attitude towards consumption was positively linked to all financial behaviours under economic strain, that is, cutting expenses, borrowing, increasing income, and gambling. Presumably, feeling deprived in terms of consumption indicates long-term financial deprivation, in which all means of surviving financially are used. Gambling, in particular, has been found to be associated with feelings of relative deprivation (Callan et al. 2008).

Finally, the hedonistic attitude towards consumption was related to the lower frequency of cutting expenses and higher frequency of borrowing, increasing income, and gambling. This aligns well with the idea that people holding hedonistic attitudes are more likely to borrow to consume at levels equal to their reference group or to increase income instead of cutting their consumption. This may stem from the fact that hedonistic attitudes promote well-being derived from the notion of “keeping up with Joneses” (Dew 2007). People with hedonistic attitudes towards consumption may also wish to derive pleasure from consumption in whatever available means (Hirschman and Holbrook 1982), instead of cutting their expenses.

### Limitations and Strengths

Some limitations need to be acknowledged concerning our study. First, although we collected data at five time points, this study was cross-sectional and precludes causal interpretations of the findings. To gain a better understanding of the process that a consumer goes through in his or her lifetime, a longitudinal study following the same participants would be ideal. Second, the study relied on self-reported data, which is prone to social desirability bias. Also, the respondents’ answers to the subjective data are open to interpretation. For example, the subjective evaluation of one’s financial situation could be studied more comprehensively in further studies (although it was not the key focus here, and it has been studied in many studies with a single measure, e.g., Ranta and Salmela-Aro 2018). Third, we did not ask about the exact situation causing economic strain in participants’ lives or whether their financial difficulties were temporary or permanent. In particular, the financial behaviour under economic strain may depend on whether financial difficulties are perceived as temporary or permanent (e.g., cutback in hours at work to a long-term disability), and the depth of the financial difficulties (e.g., due to mental or physical health, unemployment; French and Vigne 2019). In previous studies, for instance, Baek and DeVaney (2010) took into account whether and for how long a household head was unemployed during the past year and the health status of a household head. Dew and Xiao (2013) asked participants about the degree to which their finances have changed between 2008 and 2009 on a scale from one (have gotten much worse) to five (have gotten much better) and how often they worried about not being able to pay bills from one (never) to five (all the time). Unfortunately, information measuring the degree and causes of the economic strain was not collected in the current study. Instead, the survey was constructed in such a way that people were expected to answer about their situational/temporary/usual way of responding in financially tight situations. Despite this, some people might have reported about their permanent situation. Controlling for the depth of the economic strain or the situation that caused it should be considered in future research. Finally, we collected data in Finland, a Nordic welfare state. Therefore, caution regarding generalization to other countries needs to be acknowledged.

Despite the limitations, this study provides a broad picture of people’s economic behaviour under economic strain over a long period of time. Most previous studies have focused on only single dimensions of economic behaviour, such as taking loans and credit (e.g., Gamble et al. 2019; Ottaviani and Vandone 2011), gambling (e.g., Callan et al. 2008; Lusardi et al. 2011), and saving (Dwyer et al. 2011; Webley and Nyhus 2001), in one point of time. In addition, our analyses included attitudes towards consumption, which, in sociological theories, are interpreted as reflecting social status and identity (Bourdieu 1984), and hedonistic pursuit for pleasure (Campbell 1987). In our study, the attitudes appeared as powerful predictors of individuals’ financial behaviour under economic strain. This extends our understanding of consumer behaviour during macro-economic crises in particular. Consumer behaviour is not just a function of income, age, and life course stage; it contains a far more complex set of determinants.

Furthermore, our results also increase knowledge of individual motives for financial behaviour under financial strain at different age periods and thereby provide tools for financial life management (i.e., making adjustments to make ends meet), which directly relate to well-being (Baek and DeVaney 2010; Ranta et al. 2020a, b; Ranta and Salmela-Aro 2018; Serido et al. 2013; Xiao et al. 2014). Currently, a global economic crisis will emerge as a consequence of the COVID-19 virus. Therefore, there will be an urgent need for new research about changes in consumers’ behaviour under economic strain. Particularly comparative studies on the topic across societies and in different cultures will be increasingly important in the future.

### Practical Implications

The outcome of the current research, that is, increased knowledge of long-term trends in financial behaviour under economic strain in different age groups, provides valuable tools for consumer policy, consumer education, and consumer regulation. In Finland, there are very strong and organized consumer policy authorities that particularly aim at increasing consumers’ competence to manage their economy and finances (Wahlen and Huttunen 2011). Finnish authorities also strictly follow European Union consumer regulations (Raijas et al. 2010). In 2020, the Bank of Finland was given the responsibility of coordinating and implementing a national strategy for personal finance management and financial behaviour of Finnish citizens (Bank of Finland 2020).

Our research revealed that people cannot be treated as a homogeneous group of financial actors. They have different strategies in coping with economic strain, varying by age and life course stage, social background, gender, and consumption attitudes and preferences. Thus, financial regulation, consumer advice, and educational campaigns should better acknowledge these differences and flexibly target the policy actions to specific consumer groups. It is also important to be aware of the effects of the present major global trends: digitalization of consumption and finances, macro-economic fluctuations, and crises such as COVID-19 on the financial behaviour of consumers. In rapidly changing social and economic environments, consumer legislation and financial regulation will need more frequent updating in the future, both in Finland and in the European Union.

## Footnotes

1 Statistics Finland - National Accounts 2019. Retrieved from https://www.tilastokeskus.fi (accessed on 13 August 2020).

2 Retrieved from https://www.suomenpankki.fi/en/Statistics/chart-gallery/financial-cycle-indicators/private-sector-indebtedness-or-debt-servicing-burden/velkaantumisaste_ja_korkorasitus/ (accessed on 26 November 2020).

3 Retrieved from https://www.suomenpankki.fi/en/Statistics/chart-gallery/financial-cycle-indicators/private-sector-indebtedness-or-debt-servicing-burden/velkaantumisaste_ja_korkorasitus/ (accessed on 26 November 2020).

4 Retrieved from https://www.suomenpankki.fi/en/Statistics/chart-gallery/financial-cycle-indicators/private-sector-indebtedness-or-debt-servicing-burden/velkaantumisaste_ja_korkorasitus/ (accessed on 26 November 2020).

5 Retrieved from https://www.suomenpankki.fi/en/Statistics/mfi-balance-sheet/older-news/2018/vakuudettomien-kulutusluottojen-nostot-lisaantyneet-entisestaan/ (accessed on 14 November 2020).

6 Retrieved from https://www.stat.fi/til/tyti/2019/09/tyti_2019_09_2019-10-22_tie_001_en.html (accessed on 14 November 2020).
